# Lead Tolerance and Remediation Potential of Four *Indocalamus* Species in Lead-Contaminated Soil

**DOI:** 10.3390/plants13131823

**Published:** 2024-07-02

**Authors:** Jiarong Liao, Mingyan Jiang, Yangcheng Lu, Yixiong Yang, Yedan Gao, Qibing Chen, Zhenghua Luo, Xiaofang Yu

**Affiliations:** College of Landscape Architecture, Sichuan Agricultural University, Chengdu 611130, China; 15378186317@163.com (J.L.); 16602868245@163.com (Y.L.); yyx950606@163.com (Y.Y.); gyedan@163.com (Y.G.); cqb@sicau.edu.cn (Q.C.); yls@sicau.edu.com (Z.L.); xiaofangyu@sicau.edu.cn (X.Y.)

**Keywords:** Pb stress, *Indocalamus* plants, growth and physiology, Pb enrichment characteristics, tolerance

## Abstract

*Indocalamus* plants are low-growing shrubby bamboos with growth advantages, such as high biomass and strong resistance, and they are rich in germplasm resources in southern China. This study conducted soil lead (Pb) stress experiments on *Indocalamus latifolius* (Keng) McClure (LA), *Indocalamus hunanensis* B.M. Yang (HU), *Indocalamus chishuiensis* Y.L. Yang and Hsueh (CH) and *Indocalamus lacunosus* Wen (LC). Five Pb treatments (0, 500, 1000, 1500 mg·kg^−1^ Pb, and 1000 mg·kg^−1^ Pb + 1000 mg·kg^−1^ ethylenediamine tetraacetic acid (EDTA)) were established. EDTA was applied to explore the tolerance mechanism of different *Indocalamus* species after absorbing large amounts of heavy metals. The results were as follows: (1) under Pb treatment, the total relative biomass of LA, HU and LC was <100%, whereas the total relative biomass of CH was >100%; (2) after applying EDTA, the bioconcentration coefficient, translocation factor, and free proline content of the four *Indocalamus* species increased; and (3) the Pb mobility and distribution rates of the underground parts of the four *Indocalamus* species were consistently greater than those of the aboveground parts. The Pb mobility and distribution rates in the stems increased after applying EDTA, while those in the leaves decreased, as the plants tended to transfer Pb to their stems, which have lower physiological activity than their leaves.

## 1. Introduction

In the process of energy utilization, human activities, such as industrial emissions, mining, smelting, and disposal of hazardous solid waste, cause heavy metals to migrate into the atmosphere, water, and soil. Soil is the final receptor of heavy metal pollutants [1,2]. Heavy metals that remain in the soil are absorbed by plant tissues, enter the biosphere and accumulate at the nutrient level of the food chain [3]. Heavy metals enter the human body through the food chain and are transported by the blood to all parts of the body, causing adverse effects on the kidneys, liver, and vascular system [4]. Among these metals, lead (Pb) is one of the most widely and evenly distributed, and it is toxic and lethal at trace concentrations, leading to behavioral and neurological disorders in infants and children [5,6].

Compared with traditional physical and chemical remediation methods, phytoremediation is easy to implement, low cost, low labor intensity and strongly sustainable, and it is an effective and environmentally friendly method for purifying metals in soil [7,8]. Hyperaccumulator plants are ideal materials for the phytoremediation of soil contaminated by heavy metals. However, these species are rare, and they have disadvantages such as low biomass, slow growth, single metal targeting and geographical limitations [9]. In recent years, more research has focused on screening plants with high biomass, with a focus on evaluating their tolerance to heavy metals. The heavy metal tolerance of plants is mainly reflected in their growth and physiology, such as the biomass, osmotic regulation, and antioxidant systems [10,11]

In the Poaceae family, *Indocalamus*, a perennial plant of the Bambusoideae subfamily, is a shrub or small shrub, and its underground stem is of the multiaxis type. It has growth advantages, such as wide leaves, thick stems, large biomass, and strong stress resistance [12], and its resources are abundant in southern China. The vast underground whip-root system of *Indocalamus* plants is mainly distributed in the shallow soil layer, which easily absorbs and fixes metal ions and has good innate conditions for absorbing heavy metals in the soil [13]. Studies have shown that *Indocalamus decorus* Q.H. Dai exhibits strong tolerance to soil Pb stress, and Pb in its root cells has difficulty migrating (from fragments and clumps of crystals), making it a potentially useful plant for the remediation of soil Pb pollution [14,15]. However, it is not clear whether other *Indocalamus* species also exhibit the same or similar tolerances. In summary, this study was guided by the demand for high-biomass, tolerant plants for phytoremediation practices and used *Indocalamus latifolius* (Keng) McClure (LA), *Indocalamus hunanensis* B.M. Yang (HU), *Indocalamus chishuiensis* Y.L. Yang and Hsueh (CH) and *Indocalamus lacunosus* Wen (LC) as the research objects. By observing the physiological growth responses of the four *Indocalamus* species and the Pb content in their organs and other indices, the physiological growth responses and repair efficiency of these *Indocalamus* plants were investigated.

## 2. Research Content and Methods

### 2.1. Research Material

This experiment was performed at the landscape ecological experimental base of the College of Landscape Architecture, Sichuan Agricultural University (30°42′20″ N, 103°51′28″ E, altitude 530 m). The experimental site has a subtropical monsoon climate with four distinct seasons and a pleasant climate. The average annual temperature is 15.6 °C, and the average rainfall is 1004.69 mm. The LA (culms 35–65 cm tall, 0.30–0.50 cm in diameter), HU (culms 30–50 cm tall, 0.30–0.45 cm in diameter), CH (culms 30–45 cm tall, 0.25–0.35 cm in diameter) and LC (culms 30–45 cm tall, 0.25–0.35 cm in diameter) plants used in this experiment were 2-year-old ramet-shaped plants cultivated in the ecological landscape of the experimental base.

### 2.2. Research Design

Using a completely randomized block design, LA, HU, CH and LC were used as the experimental materials, and Pb served as the stress factor. Based on previous experimental designs and research results [14], four treatments were established, namely, CK (0 mg·kg^−1^ Pb), Pb500 (500 mg·kg^−1^ Pb), Pb1000 (1000 mg·kg^−1^ Pb), and Pb1500 (1500 mg·kg^−1^ Pb). In addition, ethylenediamine tetraacetic acid (EDTA), denoted as Pb1000E (1000 mg·kg^−1^ Pb + 1000 mg·kg^−1^ EDTA), was applied at a stress concentration that bamboos were found to be more tolerant to in previous research [16]. Each treatment was repeated in three pots for a total of 60 pots. EDTA is currently the most effective chelating agent found to promote the absorption of heavy metals by plants [17,18]. EDTA was applied to explore the upper limit of heavy metal enrichment in the plants and the tolerance mechanism of the plants after rapid absorption of large amounts of heavy metals. These findings were useful for further exploring the remediation potential of the studied plants.

Pb was mixed into the soil in the form of a Pb(CH_3_COO)_2_·3H_2_O salt solution, and EDTA was added to the pot in the form of a C_10_H_14_N_2_O_8_Na_2_·2H_2_O salt solution. The soil used in the experiment was air-dried mixed soil, and the configuration method involved 3:2 mixing of garden soil and nutritive soil. The basic physical and chemical properties of the mixed soils were as follows: background Pb content of 12.92 mg·kg^−1^, pH of 7.28, organic matter content of 4.38%, available nitrogen content of 162.25 mg·kg^−1^, available potassium content of 112.91 mg·kg^−1^, and available phosphorus content of 132.67 mg·kg^−1^.

The implementation and operation process of the experiment were as follows. In January 2021, the mixed soil was prepared. Pb solution was added to achieve the final soil concentration of the design concentration, and the soil was placed in a cool place for 30 days, during which the soil was mixed regularly every three days. In February 2021, 3 kg of soil was loaded into a Φ350 white plastic basin (with a chassis), and bamboo plants with strong growth and the same growth conditions were selected, with 10 bamboo plants standing in each pot. After planting, the roots were watered thoroughly, and no pests or diseases were found during the experiment. Watering and weeding were performed every three days, and the watering frequency was adjusted appropriately according to the weather conditions. Watering was performed in small amounts multiple times without overflowing the chassis, and grass was not removed from the soil inside the pot. After the *Indocalamus* species underwent the growth stages of forming shoots and growing new bamboo (from March to May), branching and leaf expansion (June), and vegetative growth (July to November), a chelating agent solution of the corresponding concentration was applied to the Pb1000E treatment once in November 2021. The plants were harvested on the 30th day after the chelating agent was applied, and the root soil samples were collected. In December 2021, the plant physiological indices, including the chlorophyll (Chl), free proline (Pro), and malonaldehyde (MDA) contents, as well as the superoxide dismutase (SOD), peroxidase (POD) and catalase (CAT) activities, were measured. The biomass of each organ (root, whip, stem, and leaf) of the plant was weighed. The total biomass was determined, and the relative biomass of each organ of each bamboo species was calculated. The unit Pb content of each organ was measured, and the Pb mobility, distribution rate, bioconcentration coefficient (BCF) and translocation factor (TF) of each vegetative organ were calculated.

### 2.3. Index Test and Method

#### 2.3.1. Measurement of Biomass

After the bamboo plants were harvested, the aboveground parts were rinsed with clean water and deionized water, and the underground parts were rinsed with clean water, 20 mmol·L^−1^ EDTA-Na_2_ solution, and deionized water. After removing excess water from the surface of the sample, the various parts of the plant were separated into the roots, rhizomes, stalks and leaves; placed in an oven at 105 °C for 30 min; and continuously dried at 80 °C to a constant weight. The specific calculation methods for the biomass were as follows: total biomass (g) = root biomass (g) + rhizome biomass (g) + stem biomass (g) + leaf biomass (g); organ relative biomass = organ biomass under Pb treatment (g) ÷ organ biomass under CK treatment × 100%; total relative biomass = total biomass under Pb treatment (g) ÷ total biomass under CK treatment × 100%. Then, the dry samples of each organ were crushed, ground, sieved, and bagged for the determination of the metal content.

#### 2.3.2. Physiological Index Determination

New leaves with a consistent growth status at 2–3 leaf positions in the middle and upper parts of each bamboo species were collected from four directions in each pot, cut and mixed after being gently wiped clean with gauze. Then, 0.2 g of each sample was weighed, wrapped in tin foil paper, frozen in liquid nitrogen, and stored at −80 °C for the determination of the various physiological indices. The Chl content was extracted using the acetone extraction method [19]. The SOD activity was determined using the nitroblue tetrazolium photochemical reduction method [20]. The POD activity was measured via the guaiacol method [21]. The CAT activity was measured using ultraviolet spectrophotometry [22]. The MDA content was determined using the thiobarbituric acid method [23]. The Pro content was determined using the acidic ninhydrin colorimetric method [24].

#### 2.3.3. Determination of Metal Content in Plants

After the harvested plant samples were dried to a constant weight, they were ground and passed through a 100-mesh sieve with a plastic sealing pocket to be stored for testing. A 0.2 g sample was dissolved in nitric acid (5 mL HNO_3_, 1 mL HClO_4_) at 80 °C, 100 °C, 120 °C, 140 °C, 160 °C, and 180 °C for 20 min, 1 h, 1 h, 1 h, 1 h, and 2 h, respectively, for digestion. The boiled liquid was washed into a 50 mL volumetric bottle with 0.5% dilute nitric acid and then filtered into a 50 mL plastic bottle. Finally, a corresponding metal standard curve for the gradient concentration was created, and the metal content of the tissues was determined using an atomic absorption flame spectrophotometer (Shimadzu AA-7000, Kyoto, Japan). At the same time, the Pb accumulation and Pb mobility and distribution rate in the organs were calculated. The specific methods were as follows:

Pb accumulation in organs (μg) = Pb content in organs (mg/kg) × organ biomass (μg)

Total Pb accumulation in plants (μg) = Pb accumulation in roots (μg) + Pb accumulation in rhizomes (μg) + Pb accumulation in stems (μg) + Pb accumulation in leaves (μg)

Pb mobility and distribution rate in organs (100%) = Pb accumulation in organs (μg) ÷ total Pb accumulation in plants (μg) × 100%

In addition, BCF and TF were calculated based on the organ biomass and the Pb content in the organs. The specific method was as follows [25]:

Pb content in plants = total Pb accumulation in plants (mg)/total biomass (kg)

BCF = Pb content in plants (mg/kg) ÷ Pb content in soil (mg/kg)

TF = Pb content in the aboveground parts of plants (mg/kg) ÷ Pb content in underground parts of plants (mg/kg)

### 2.4. Data Statistics and Analysis Methods

Excel 2010 was used for the data calculation and chart production. All of the data were analyzed using SPSS 23.0 software for a one-way analysis of variance at a level of *p* < 0.05. Bivariate correlation analysis was performed at *p* < 0.05 and *p* < 0.01.

## 3. Results and Analysis

### 3.1. Growth and Physiological Characteristics of the Four Indocalamus Species

#### 3.1.1. Organ Biomass Allocation of the Four *Indocalamus* Species

**Figure 1:**
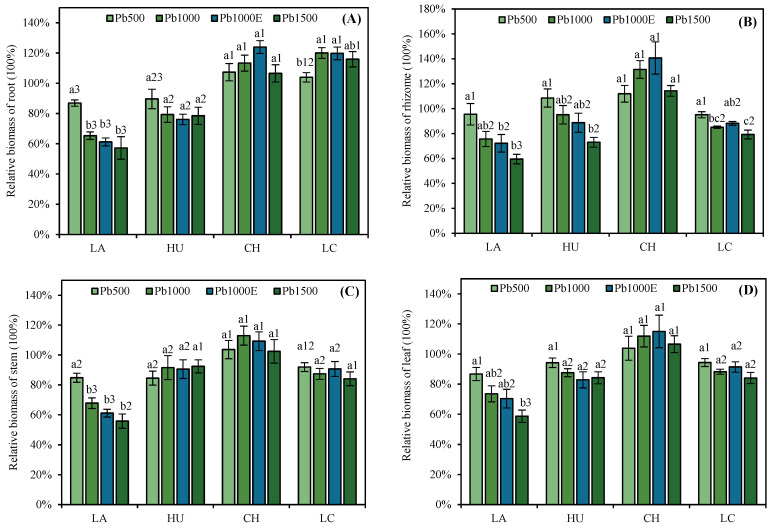
Effect of soil lead (Pb) stress on the relative biomass of roots (**A**), rhizomes (**B**), stems (**C**), and leaves (**D**) of *Indocalamus* species. All of the data are presented as the mean ± standard error (*n* = 3). Different lowercase letters indicate significant differences between different soil Pb concentrations for the same bamboo species (Duncan, *p* < 0.05), and different numbers indicate significant differences between different bamboo species under the same soil Pb concentration (Duncan, *p* < 0.05).

Figure 1 shows that under Pb treatment, the relative biomass of the roots of LA and HU was <100%. In contrast, the relative biomass of the roots of CH and LC was >100%, and their root biomass was greatest under the Pb1000 treatment. In addition, under Pb treatment, the biomasses of the rhizome, stem, and leaf of CH were >100% and were consistently greater than those of the other three bamboo species. There was no significant difference in the relative biomass of the roots, rhizomes, stems, or leaves of the four *Indocalamus* species in the Pb1000E treatment group compared with the Pb1000 treatment group (*p* > 0.05).

According to Table 1, the total relative biomass of the four *Indocalamus* species decreased in the following order: CH > LC > HU > LA. The total relative biomass of CH was always >100%. The total relative biomass of LC, HU, and LA decreased with an increasing stress concentration, and that of LA showed the greatest decrease.

#### 3.1.2. Physiological Characteristics of the Four *Indocalamus* Species

**Figure 2:**
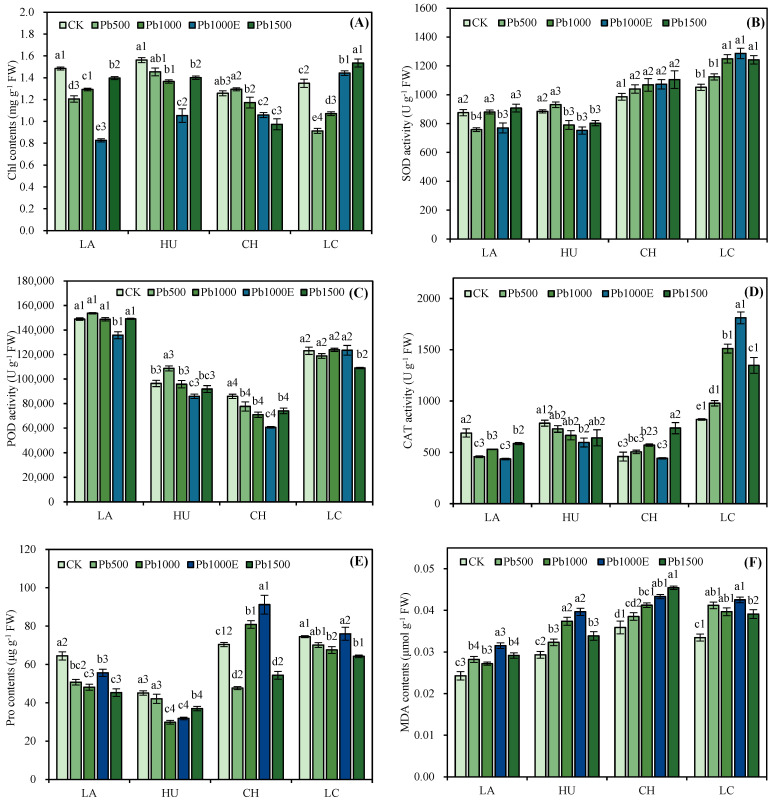
Effects of soil Pb stress on the chlorophyll content (**A**), superoxide dismutase activity (**B**), peroxidase activity (**C**), catalase activity (**D**), free proline content (**E**), and malondialdehyde content (**F**) of *Indocalamus* species. All of the data are presented as the mean ± standard error (*n* = 3). Different lowercase letters indicate significant differences between different soil Pb concentrations for the same bamboo species (Duncan, *p* < 0.05), and different numbers indicate significant differences between different bamboo species under the same soil Pb concentration (Duncan, *p* < 0.05).

According to Figure 2, under Pb treatment, the Chl contents of LA, HU, and CH did not significantly differ or were lower than those of CK. The Chl content in the LC treatment was significantly greater than that in the CK treatment under Pb1500, with an increase of 13.74%. Under the Pb500, Pb1000, and Pb1500 treatments, the SOD and CAT activities of CH and LC were greater than those of CK, the POD activities of CH and LC were lower than those of CK or were not significantly different from those of CK, and the CAT activity of LC increased by 19.32%, 84.41%, and 64.41%, respectively, compared to CK. Under the Pb500, Pb1000, and Pb1500 treatments, the Pro contents of LA, HU, and LC were lower than that of CK. Under the Pb500 and Pb1500 treatments, the Pro content of CH was significantly lower than that of CK, with decreases of 32.25% and 22.77%, respectively. Under the Pb1000E treatment, the Pro and MDA contents of LA, HU, CH, and LC all increased compared to those under the Pb1000 treatment, with LA showing the greatest increase, at 15.58% and 15.90%, respectively.

### 3.2. Pb Accumulation and Transport Characteristics of the Four Indocalamus Species

#### 3.2.1. Pb Content in the Organs of Four *Indocalamus* Species

**Figure 3:**
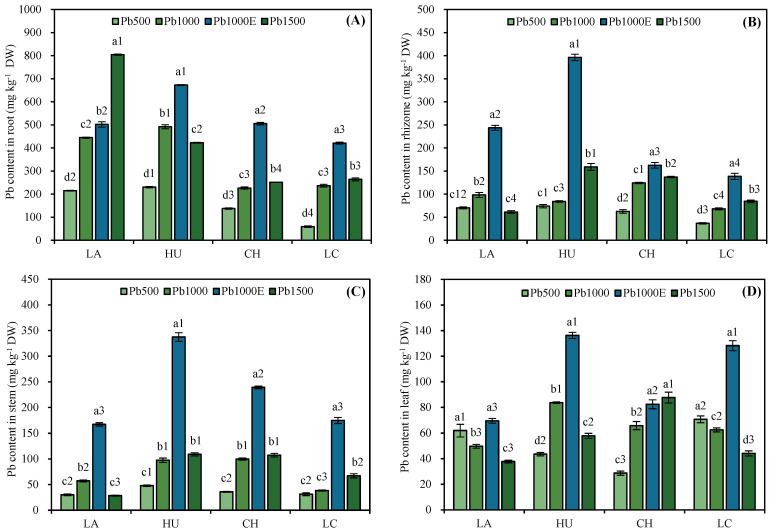
Effects of soil Pb stress on the Pb content in roots (**A**), rhizomes (**B**), stems (**C**), and leaves (**D**) of *Indocalamus* species. All of the data are presented as the mean ± standard error (*n* = 3). Different lowercase letters indicate significant differences between different soil Pb concentrations for the same bamboo species (Duncan, *p* < 0.05), and different numbers indicate significant differences between different bamboo species under the same soil Pb concentration (Duncan, *p* < 0.05). Under CK (Pb-free treatment), due to the low Pb content in various organs of *Indocalamus*, the instrumental detection values were 0.

According to Figure 3, under the Pb500, Pb1000, and Pb1500 treatments, the Pb content in the roots of LA, CH, and LC increased with an increasing stress concentration, whereas the Pb content in the roots of HU tended to first increase and then decrease. Under the Pb1500 treatment, the Pb content in the roots of LA was significantly greater than that in the roots of the other three bamboo species and was 1.90, 3.20, and 3.05 times greater than that in the roots of HU, CH, and LC, respectively. Under the Pb500, Pb1000, and Pb1500 treatments, the Pb content in the rhizome and stem of LA tended to first increase and then decrease with an increasing stress concentration, and the Pb content in the rhizome and stem of HU, CH, and LC increased. Regarding the Pb content in the leaves, the Pb content in the leaves of LA and LC tended to decrease with an increasing stress concentration, that of HU tended to first increase and then decrease, and that of CH tended to increase.

In addition, under the Pb1000E treatment, the Pb content in the roots, rhizomes, stems, and leaves of the four *Indocalamus* species increased compared to that under the Pb1000 treatment. Under the Pb1000E treatment, the Pb content in the roots of CH increased the most (123.24%), the Pb content in the rhizome of HU increased the most (370.79%), and the Pb content in the stems and leaves of LC increased the most (354.88% and 105.47%, respectively) compared with those under the Pb1000 treatment.

#### 3.2.2. Pb Accumulation and Transport in the Four *Indocalamus* Species

**Figure 4:**
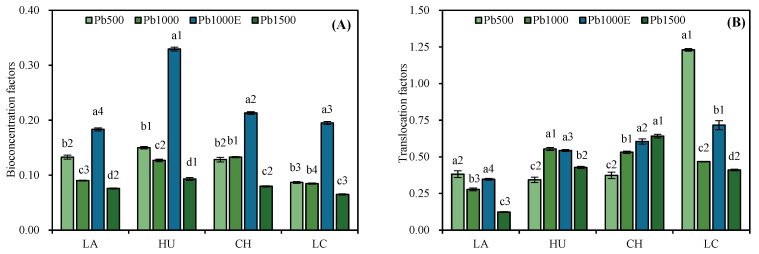
Bioconcentration factors (**A**) and translocation factors (**B**) of *Indocalamus* species under soil Pb stress. All of the data are presented as the mean ± standard error (*n* = 3). Different lowercase letters indicate significant differences between different soil Pb concentrations for the same bamboo species (Duncan, *p* < 0.05), and different numbers indicate significant differences between different bamboo species under the same soil Pb concentration (Duncan, *p* < 0.05). Under CK (Pb-free treatment), the instrumental detection values of the Pb content in various organs of *Indocalamus* plants were 0, so they are not shown in the figure.

Figure 4 shows that the BCF of the four *Indocalamus* species under the Pb1500 treatment was lower than under the Pb1000 treatment and Pb500 treatment. Under the Pb1000 treatment, the application of EDTA increased the BCF of the four *Indocalamus* species by 60.39–159.52%.

Under the Pb500, Pb1000, and Pb1500 treatments, the largest TF values were found in LC (1.23), HU (0.55), and CH (0.64), respectively. After the application of EDTA, the TF of the other three *Indocalamus* species except HU increased significantly, increasing by 13.2–52.92%.

According to Table 2, under the Pb500, Pb1000, and Pb1500 treatments, the Pb mobility and distribution rate in the underground parts (roots and rhizomes) of the four *Indocalamus* species were consistently greater than those in the aboveground parts (stems and leaves). Under the Pb1000E treatment, the Pb mobility and distribution rates in the aboveground parts of the four *Indocalamus* species increased compared to those under the Pb1000 treatment, and the Pb mobility and distribution rates in the stems increased (the increase range was 4.84–8.38%). In contrast, the Pb mobility and distribution rate in the leaves decreased (the range of decrease was 0.04–6.02%).

### 3.3. Correlation Analysis of the Tolerances of the Four Indocalamus Species

#### 3.3.1. Correlations between Growth and Pb Content of the Four *Indocalamus* Species

As shown in Table 3, the root biomass of LA and HU was significantly negatively correlated with the Pb content, whereas the root biomass of CH and LC was significantly positively correlated with the Pb content.

#### 3.3.2. Correlations between Physiological Indices and Pb Contents in the Leaves of the Four *Indocalamus* Species

According to Table 4, the MDA content of the four *Indocalamus* species was highly significantly positively correlated with the Pb content in the leaves, whereas the Chl content of LA, HU, and CH was highly significantly negatively correlated with the Pb content in the leaves. Furthermore, the SOD activity, CAT activity, and Pro content of LA and HU were significantly/extremely significantly negatively correlated with the Pb content in the leaves, whereas the SOD activity of CH and LA was significantly/extremely significantly positively correlated with the Pb content in the leaves.

## 4. Discussion

In response to heavy metal stress, the first line of defense on the part of plants is to reduce the absorption of metals, limiting the entry of metals into cells [26]. Zhang et al. [27] reported that as the concentration of heavy metals increased, the heavy metal content in the roots of *Lolium perenne* L. increased. Xu et al. [28] reported that the Pb content in hemp gradually increased with an increasing soil Pb concentration. In this study, with an increasing stress concentration, the Pb content in the roots of LA, CH, and LC increased. However, the BCF of the four *Indocalamus* species under the Pb1500 treatment was lower than under the Pb1000 treatment and Pb500 treatment. Under high-concentration soil Pb stress, the four *Indocalamus* species alleviated the stress caused by Pb by reducing their Pb accumulation [29].

Most plants effectively fix heavy metals that have already entered the plants through their roots to alleviate the damage caused by heavy metal stress to other organs [30]. To avoid damage to photosynthetic tissue induced by Pb stress, *Lonchocarpus cultratus* plants mainly accumulate Pb in their roots (67–99%; Oliveira et al. [31]), and the roots of *Vetiveria zizanioides* absorb more Pb than the shoots [32]. In addition, Cai et al. [14] reported that under soil Pb stress, *Indocalamus decorus* Q.H. Dai mainly fixes Pb in the stem and rhizome, which is a key tolerance strategy. In this study, large amounts of Pb were fixed in the roots and rhizomes of the four *Indocalamus* species, reducing the toxicity to the plants by preventing the transfer of heavy metals from the underground to the aboveground parts [33].

In addition, most studies have shown that the application of EDTA can improve the accumulation and transport capacity of heavy metals in plants. Mousavi et al. [34] reported that the application of EDTA promoted the accumulation of cadmium in the aboveground and root parts of *Abelmoschus esculentus* L., and Wang et al. [35] reported that the application of EDTA promoted the translocation of Pb to the upper part of *Bidens maximowicziana*. In this study, after the application of EDTA, the TF of LA, HU, and CH increased, and the heavy metal content in the stems and leaves of the four *Indocalamus* species increased. The Pb mobility and distribution rates in the stems increased, whereas the Pb mobility and distribution rates in the leaves decreased. It can be concluded that *Indocalamus* species tend to transfer Pb to the stems, which have lower physiological activity than the leaves, to reduce the damage caused by heavy metals to the leaves and to maintain normal physiological activity and photosynthesis.

Moreover, the second line of defense of plants against heavy metal stress involves the adoption of tolerance strategies to address the toxicity of accumulated metal ions after they enter the cytoplasm [36]. The tolerance strategies of plants against heavy metal stress include isolating heavy metals in vacuoles and synthesizing phytochelatins [37]. When the above strategies are insufficient to inhibit damage, plant cells will trigger the production of reactive oxygen species (ROS), which may lead to significant oxidative stress [38]. In this study, the MDA content of the four *Indocalamus* species showed a highly significant positive correlation with the Pb content in the leaves. The change in the MDA content in the leaves is considered an indicator of the physiological response of plants to heavy metal stress [39]. As the accumulation of Pb in the leaves increased, the damage to the *Indocalamus* species also increased. At this point, cells activate the ROS clearance mechanism by producing antioxidant compounds and increasing antioxidant enzyme activity [40]. In our study, under soil Pb stress, CH and LC effectively increased their SOD and CAT activities. This result was similar to that of *Populus nigra* ‘Italica’, in which the SOD and CAT activities increased under heavy metal stress [41], effectively clearing the excess hydrogen peroxide and protecting the plants from oxidative damage caused by heavy metal exposure. Pro is an effective osmotic regulator that reduces the oxidative stress caused by heavy metal stress by clearing free radicals [42,43]. In our study, the Pro content of the four *Indocalamus* species decreased to varying degrees under soil Pb stress, which may have been due to the inhibition of Pro synthesis or accelerated decomposition metabolism [44]. The Pro content of the four *Indocalamus* species increased with an increasing Pb content in their leaves after the application of EDTA, which was similar to the increase in the Pro content observed in *Helianthus annuus* L. after the application of EDTA [45], indicating that all four *Indocalamus* species can maintain cell homeostasis by increasing the Pro content when the stress effect of heavy metals is high [46].

Among plant organs, the growth of roots is considered an important parameter for determining tolerance to metal stress [47]. The relative biomass of the CH and LC roots under Pb treatment was greater than that under CK treatment in our study. This effect may weaken the toxic effects of heavy metals by increasing the root biomass, which is an adaptive behavior to soil Pb stress [48]. The growth of various organs of CH was not significantly inhibited (Figure S1), and its relative biomass was greater than 100% under all the treatments, indicating strong adaptability to soil Pb stress.

In addition, unlike EDTA, which reduced the biomass of *Pelargonium hortoum* seedlings by 28.4% under soil Pb stress [49], the relative biomass of each organ of the four *Indocalamus* species was not significantly affected by EDTA application in this study, and the BCF and TF also increased. It is hypothesized that this difference is due to the timing of the EDTA application, which was not during the vigorous growth period of the *Indocalamus* species. It was in the process of nutrient accumulation and was about to hibernate for winter. Most scholars believe that EDTA is not sufficient to effectively reduce the heavy metal content in soil (via the decrease in plant biomass) and can cause heavy metal leaching, increasing the risk of environmental pollution [50,51,52]. The use of chelating agents is an effective means of improving the ability of plants to absorb heavy metals, but the impact on plant growth and secondary pollution of the environment should be considered. In this study, the Pb content in various organs of *Indocalamus* species increased by up to 123.24% (roots; CH), 370.79% (rhizomes; HU), 354.88% (stems; LC), and 105.47% (leaves; LC) after EDTA was applied. The growth of the *Indocalamus* species did not significantly change after rapid absorption of a large amount of heavy metals, and these four species exhibited a high tolerance to soil Pb stress.

## 5. Conclusions

In this study, the BCF and TF of the four *Indocalamus* species were all <1. Thus, these plants were not hyperaccumulators but had good tolerance to soil Pb stress. The four *Indocalamus* species mainly store Pb in their underground parts (roots and rhizomes). After the application of EDTA, the Pb content in the organs of the four *Indocalamus* species increased, with increases of up to 370.79% (rhizome) and 354.88% (stem); thus, rhizomes and stems are potential organs for storing heavy metals. In addition, when Pb is transported to the aboveground parts of the plants, the four *Indocalamus* species tend to store Pb in their stems, which have lower physiological activity than their leaves, to maintain normal photosynthesis in the leaves. Compared with those of the other bamboo species, the biomass of various organs of CH increased under Pb stress, indicating that CH has strong adaptability to soil Pb stress and strong phytoremediation potential. However, this study only involved four *Indocalamus* species and included only one bamboo plant growth cycle. In the future, long-term experiments are needed to reveal the response of the biomass allocation and morphogenesis of *Indocalamus* plants to soil Pb stress. In addition, more comparative studies of the Pb tolerance of *Indocalamus* plants are needed to explore the application potential of *Indocalamus* plants in Pb pollution remediation practices.

## Figures and Tables

**Table 1:** Total relative biomass of four *Indocalamus* species under soil Pb stress.

Treatments	Bamboo Species			
	LA	HU	CH	LC
Pb500	88.73% ± 1.21% ^a1^	95.41% ± 4.46% ^a1^	108.49% ± 5.28% ^a1^	95.59% ± 0.85% ^a1^
Pb1000	71.57% ± 1.31% ^ab2^	90.16% ± 4.54% ^ab2^	121.28% ± 5.13% ^a1^	90.62% ± 1.39% ^bc2^
Pb1000E	67.23% ± 3.33% ^b2^	86.15% ± 2.72% ^ab2^	126.51% ± 8.96% ^a1^	93.32% ± 1.58% ^ab2^
Pb1500	57.93% ± 1.85% ^b3^	82.33% ± 1.38% ^b2^	109.72% ± 2.49% ^a1^	85.75% ± 1.55% ^c2^

All of the data are presented as the mean ± standard error (*n* = 3). Different lowercase letters indicate significant differences between different soil Pb concentrations for the same bamboo species (Duncan, *p* < 0.05), and different numbers indicate significant differences between different bamboo species under the same soil Pb concentration (Duncan, *p* < 0.05).

**Table 2:** Pb mobility and distribution rate of *Indocalamus* species under soil Pb stress (100%).

Bamboo Species	Treatment	Plant Organs				Aerial Part	Underground Part
		Root	Rhizome	Stem	Leaf		
LA	Pb500	33.62% ± 0.24% ^c12^	29.43% ± 0.82% ^b3^	10.87% ± 0.58% ^c2^	26.09% ± 20.5% ^a1^	36.95% ± 2.25% ^a1^	63.05% ± 1.07% ^c23^
	Pb1000	43.84% ± 0.27% ^b1^	27.33% ± 1.53% ^b3^	13.90% ± 0.46% ^b3^	14.93% ± 0.44% ^b1^	28.83% ± 0.82% ^b2^	71.17% ± 1.76% ^b2^
	Pb1000E	27.67% ± 0.63% ^d3^	38.64% ± 0.83% ^a1^	21.79% ± 0.43% ^a2^	11.91% ± 0.32% ^bc2^	33.70% ± 0.59% ^a2^	66.30% ± 1.11% ^c2^
	Pb1500	71.15% ± 0.24% ^a1^	13.74% ± 0.67% ^c4^	5.83% ± 0.15% ^d4^	9.28% ± 0.25% ^c2^	15.11% ± 0.40% ^c4^	84.89% ± 0.56% ^a1^
HU	Pb500	34.63% ± 0.45% ^b1^	34.87% ± 1.78% ^a23^	16.08% ± 0.50% ^d1^	14.42% ± 0.43% ^b2^	30.50% ± 0.92% ^c2^	69.50% ± 1.87% ^a2^
	Pb1000	40.50% ± 0.70% ^a2^	21.53% ± 0.43% ^c4^	22.01% ± 0.94% ^c1^	15.95% ± 0.11% ^a1^	37.96% ± 0.88% ^ab1^	62.04% ± 0.27% ^bc3^
	Pb1000E	21.48% ± 0.06% ^c4^	38.20% ± 0.65% ^a1^	30.40% ± 0.75% ^a1^	9.93% ± 0.18% ^c3^	40.32% ± 0.59% ^a1^	59.68% ± 0.59% ^c3^
	Pb1500	34.08% ± 0.14% ^b3^	30.88% ± 1.42% ^b3^	24.54% ± 0.69% ^b1^	10.50% ± 0.36% ^c2^	35.04% ± 1.05% ^b1^	64.96% ± 1.56% ^b3^
CH	Pb500	32.25% ± 0.65% ^a2^	44.21% ± 2.80% ^a1^	12.43% ± 0.33% ^c2^	11.11% ± 0.64% ^b2^	23.54% ± 0.34% ^c3^	76.46% ± 3.42% ^a1^
	Pb1000	25.02% ± 0.51% ^b3^	45.98% ± 0.52% ^a1^	16.79% ± 0.35% ^b2^	12.21% ± 0.59% ^b2^	29.00% ± 0.43% ^b2^	71.00% ± 0.18% ^ab2^
	Pb1000E	33.91% ± 0.37% ^a2^	35.73% ± 1.30% ^b1^	21.62% ± 0.24% ^a2^	8.73% ± 0.37% ^c4^	30.36% ± 0.53% ^ab3^	69.64% ± 1.25% ^b12^
	Pb1500	25.56% ± 0.11% ^b4^	43.20% ± 0.50% ^a1^	16.06% ± 0.53% ^b2^	15.18% ± 0.74% ^a1^	31.24% ± 0.77% ^a2^	68.76% ± 0.48% ^b2^
LC	Pb500	18.31% ± 0.99% ^c3^	41.04% ± 1.64% ^a12^	10.87% ± 0.94% ^b2^	29.78% ± 1.12% ^a1^	40.65% ± 0.75% ^a1^	59.35% ± 1.28% ^c3^
	Pb1000	44.58% ± 1.11% ^a1^	35.80% ± 1.15% ^b2^	6.67% ± 0.19% ^c4^	12.96% ± 0.32% ^b2^	19.63% ± 0.48% ^c3^	80.37% ± 2.26% ^a1^
	Pb1000E	37.05% ± 0.39% ^b1^	35.28% ± 1.71% ^b1^	14.75% ± 0.50% ^a3^	12.92% ± 0.39% ^b1^	27.67% ± 0.66% ^b4^	72.33% ± 1.72% ^b1^
	Pb1500	43.95% ± 1.14% ^a2^	37.77% ± 1.22% ^ab2^	10.28% ± 0.62% ^b3^	8.00% ± 0.36% ^c3^	18.28% ± 0.30% ^c3^	81.7% ^2^ ± 1.28% ^a1^

All data are the mean ± standard error (*n* = 3). Different lowercase letters mean significant differences between different soil Pb concentrations for the same bamboo species (Duncan, *p* < 0.05), and different numbers mean significant differences between different bamboo species under the same soil Pb concentrations (Duncan, *p* < 0.05). Under CK (Pb-free treatment), the instrumental detection values of the Pb content in various organs of *Indocalamus* plants were 0, so they are not shown in the table.

**Table 3:** Correlations between biomass and Pb content in each organ of the *Indocalamus* species.

Organs	Bamboo Species			
	LA	HU	CH	LC
Root	−0.875 **	−0.757 **	0.713 **	0.764 **
Rhizome	−0.405	−0.330	0.698 **	−0.613 *
Stem	−0.548 *	−0.153	0.267	−0.208
Leaf	−0.470	−0.667 **	0.366	−0.291

* is significantly correlated at the 0.05 level (both sides), and ** is extremely significantly correlated at the 0.01 level (both sides).

**Table 4:** Correlations between physiological indices and Pb contents in the leaves of the *Indocalamus* species.

Physiological Indexes	Bamboo Species			
	LA	HU	CH	LC
Chlorophyll content	−0.795 **	−0.925 **	−0.830 **	−0.028
Superoxide dismutase activity	−0.588 *	−0.723 **	0.551 *	0.653 **
Peroxidase activity	−0.315	−0.527 *	−0.751 **	0.185
Catalase activity	−0.941 **	−0.615 *	0.499	0.798 **
Free proline content	−0.519 *	−0.826 **	0.275	0.245
Malondialdehyde contents	0.802 **	0.928 **	0.893 **	0.837 **

* is significantly correlated at the 0.05 level (both sides), and ** is extremely significantly correlated at the 0.01 level (both sides).

## Data Availability

The datasets used or analyzed during the current study are available from the corresponding author on reasonable request.

## Supplementary Materials

The following supporting information can be downloaded at https://www.mdpi.com/article/10.3390/plants13131823/s1, Figure S1 Effect of soil lead (Pb) stress on the biomass of roots (A), rhizomes (B), stems (C), and leaves (D) of *Indocalamus* species.
