# Supplementary material for: Lead Tolerance and Remediation Potential of Four Indocalamus Species in Lead-Contaminated Soil
Source: Plants (Basel). 2024 Jul 2;13(13):1823. doi: 10.3390/plants13131823 (PMC11244322; doi:10.3390/plants13131823)
Supplement: Supplementary file 1 [file plants-13-01823-s001.zip › plants-2979086-supplementary.pdf]

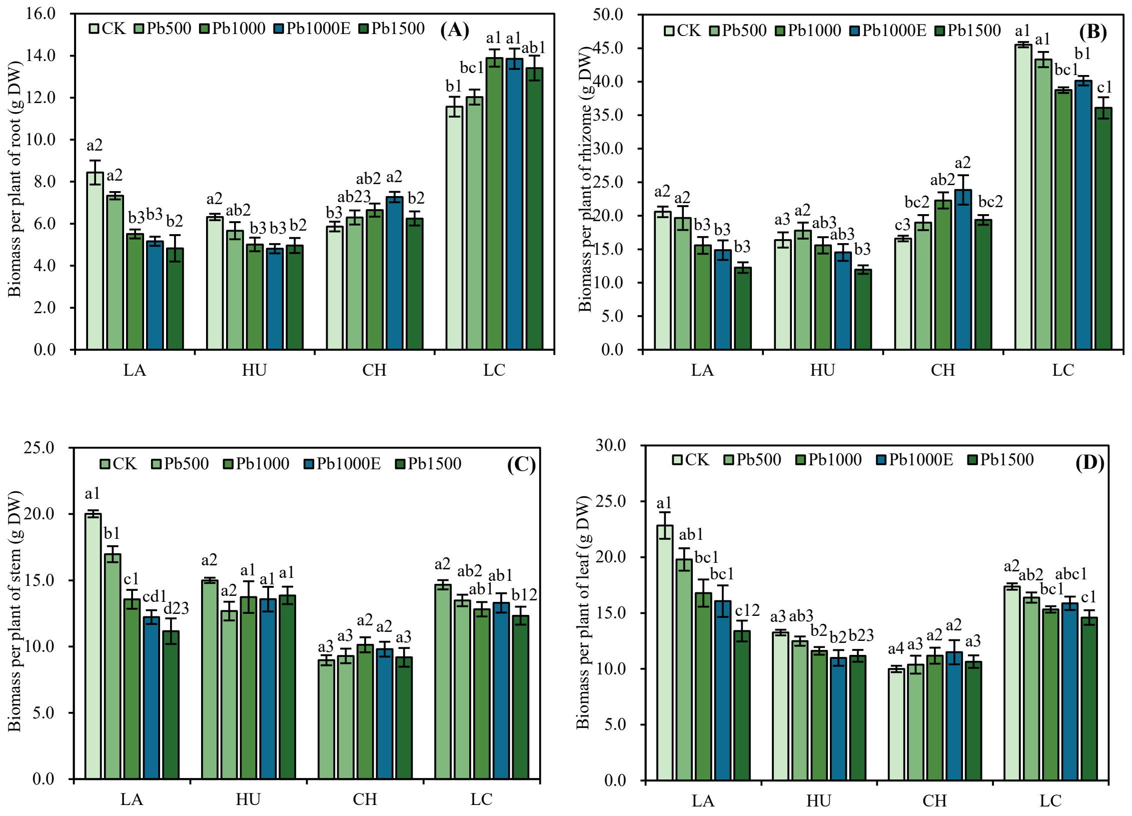

Figure S1 Effect of soil lead (Pb) stress on the biomass of roots (A), rhizomes (B), stems (C), and leaves (D) of *Indocalamus* species. All of the data are presented as the mean ± standard error (n = 3). Different lowercase letters indicate significant differences between different soil Pb concentrations of the same bamboo species (Duncan, $p<0.05$), and different numbers indicate significant differences between different bamboo species under the same soil Pb concentration (Duncan, $p<0.05$).
